# The influence of psychological resilience and nursing practice environment on nurses' moral courage: A cross‐sectional study

**DOI:** 10.1002/nop2.2163

**Published:** 2024-04-20

**Authors:** Zhang Ruixin, He Shan, Tang Yongli, Jie Chen, Chen Qianzhu, Wang Xue

**Affiliations:** ^1^ Department of Orthopaedics The First Affiliated Hospital of Chongqing Medical University Chongqing China; ^2^ Orthopedic Laboratory of Chongqing Medical University Chongqing China; ^3^ School of Nursing Chongqing Medical University Chongqing China

**Keywords:** moral courage, nurses, nursing practice environment, psychological resilience

## Abstract

**Aim:**

To determine the relationship between psychological resilience, nursing practice environment, and moral courage of clinical nurses and also the factors influencing moral courage.

**Design:**

Cross‐sectional study.

**Methods:**

586 nurses from a general hospital were selected by convenience sampling method in January 2023. The general information questionnaire, Nurses' Moral Courage Scale (NMCS), Resilience Scale, and Practice Environment Scale (PES) were measured. Hierarchical linear regression analysis was used to explore the influencing factors of clinical nurses' moral courage.

**Results:**

Nurses' average moral courage score was 79.00 (69.00, 91.00). The nurses' moral courage was positively correlated with psychological resilience and nursing practice environment. Multivariate linear regression analysis showed that psychological resilience and nursing practice environment entered the regression equation, accounting for 23.4% of the total variation. Psychological resilience and nursing practice environment are the main factors affecting the moral courage of clinical nurses. Nursing managers should conduct moral courage training, develop a decent nursing practice environment, pay attention to the psychological emotions of nurses, and actively build a safe, open, and supportive atmosphere for moral behaviour.

## 1 INTRODUCTION

To provide high‐quality nursing services and ensure the sustainable development of the nursing profession, nurses face many ethical issues; at the same time, they are required to be morally competent in their professional performance (Luo et al., 2023). Moral courage is defined as an individual's ability to persist in doing the right thing based on moral principles in the face of knowing risks, even if the consequences are negative (Dinndorf‐Hogenson, 2015; Rakhshan et al., 2021). Previous studies have shown that moral courage is closely related to clinical nursing quality, patient safety, and nurse–patient relationship (Huang et al., 2023; Lucas et al., 2023). Nurses with good moral courage can fulfil their professional commitment, make correct decisions and behaviours in moral dilemmas, and better embody the connotation of quality nursing, such as improving the quality of nursing, improving the quality of life of patients, and improving patient safety and satisfaction (Pajakoski et al., 2021). In addition, moral courage also affects the personal development of nurses. The literature indicates that the lack of moral courage will reduce the professional identity of nurses, resulting in job burnout, moral apathy, mental distress, and even turnover, which ultimately leads to a shortage of human resources (Berdida, 2023; Giannetta et al., 2022; Kleemola et al., 2020). The potential and widespread future impact of moral courage on nurses, patients, and the health care system reinforces the need to focus on the level of moral courage.

## 2 BACKGROUND

Varied studies reported that the moral courage level of nurse in the clinical setting differed. Nurses' moral courage was relatively high at a major university hospital in Southern Finland (mean = 4.09) (Hauhio et al., 2021). However, in the Chinese medical environment, it was at a low level in the study by Hong et al. (2023), which may be related to the internal factors of nurses themselves and the external environment they were exposed to, and these factors hinder the courageous behaviour of nurses. It is vital to fully understand the influencing factors of moral courage of clinical nurses and formulate relevant policies and interventions.

In the tripartite levels of courage (TLC) model by Rate (2007), the inner ring is the core factor, which includes external circumstances, motivation towards excellence, and volition/resilience; the central ring is the contingency factor, which include affect/emotion and cognitive processes; and the outer loop is the peripheral factor, which includes demographic variables, social cognitive bias, and success and failure experience. The research has primarily focused on identifying these components. Core factors are important reasons to stimulate courage, contingency factors are highly correlated with courage, and peripheral factors can influence our view of courage.

Among these factors, individuals with resilience can successfully recover from or cope with hardship. It is a dynamic process of individuals' good adaptation in a harmful environment and a type of aptitude or characteristic of individuals (Luthar et al., 2000). Several studies have shown that a statistically significant positive relationship was found between nurses' psychological resilience and moral courage, and a higher level of psychological resilience could indicate a higher level of moral courage (Khoshmehr et al., 2020; Oshio et al., 2018). As an important protective factor of moral issues, psychological resilience can reduce the occurrence and impact of moral dilemmas, or at least avoid their negative consequences by supporting their moral dynamic role, so that nurses can reconstruct patient care and promote their moral behaviour (Heinze et al., 2021). Moreover, as an important factor in the work of nurses, the nursing practice environment is defined as the organizational characteristics of the work environment that enhance or limit professional nursing practice, enabling nurses to gain more autonomy, responsibility, and control of their work (Jingxia et al., 2022; Lake, 2002). A favourable practice environment or organizational care can boost nurses' moral courage (Sadooghiasl et al., 2018).

Prior researches on moral courage were about scale development (Numminen et al., 2019), status surveys (Khoshmehr et al., 2020; Numminen et al., 2019), factor exploration (Abdollahi et al., 2021; Pakizekho & Barkhordari‐Sharifabad, 2022; Pirdelkhosh et al., 2022), managerial intervention (Abbasi et al., 2019; Hutchinson et al., 2015; Khoshmehr et al., 2020), and other fields. Nevertheless, the investigation of related factors in our country is still insufficient, and there are no relevant reports on the impact of psychological resilience and the nursing practice environment on moral courage. Therefore, the purpose of this study was to investigate the status of clinical nurses' moral courage and to analyse the influence of inner factors in the TLC model, namely psychological resilience and nursing practice environment, on moral courage, in order to provide some reference for the formulation of effective measures to alleviate nurses' moral dilemmas and to increase their level of moral courage.

## 3 METHODS

### 3.1 Design and participants

In the present study, an online cross‐sectional design was applied, and convenience sampling was used to select clinical nurses as samples from a general hospital with >3000 nurses and > 3000 beds in Chongqing, China. The study was conducted from 10 January to 31 January 2023. The inclusion criteria were as follows: (1) Registered Nurse; (2) work experience ≥12 months; and (3) willing to participate in the study and signed an informed consent form. The exclusion criteria were as follows: (1) training nurses, programme nurses, and trainees and (2) persons on sick leave or personal leave during the survey period.

The research items consist of 10 items of general information, 4 dimensions of the Nurses' Moral Courage Scale (NMCS), 4 dimensions of the Resilience Scale, and 5 dimensions of the Practice Environment Scale (PES), including a total of 23 statistical analysis variables. Using the Kendall sample size estimation method (Zhang et al., 2020), the sample size estimation formula *N* = [Max (number of variables)]*20, plus a 20% invalid sample size. Therefore, a sample size of 20 times the number of variables was selected (i.e. 460). Considering the inaccuracy and inefficiency, the sample size was increased by 20% to 552 samples.

### 3.2 Measurement

#### 3.2.1 Demographic characteristics

The general information questionnaire has 10 variables, including gender, age, work experience, whether a specialist nurse, professional title, employment form, the prime shift in the past 6 months, the highest degree, whether they had children, and whether they had participated in moral courage training.

#### 3.2.2 Nurses' Moral Courage Scale

Nurses' moral courage was assessed using the Chinese version of NMCS. It was developed by Numminen et al. (2019) to measure the self‐assessed moral courage of clinical nurses with Cronbach's α coefficient of 0.93 for the questionnaire overall and 0.73 ~ 0.82 for each dimension. The Chinese version of NMCS was translated by Wang et al. (2019). The scale comprised 4 dimensions, including commitment to good care (5 items), compassion and true presence (5 items), moral integrity (7 items), and moral responsibility (4 items) with a total of 21 items. Each item was rated on a five‐point Likert scale (1 = completely inconsistent with me to 5 = completely consistent with me). The total score of the scale is 21 ~ 105 points, and the higher the score, the higher the level of nurses' moral courage. Cronbach's α coefficient of the Chinese version of NMCS was 0.905 for the total scale and 0.778 ~ 0.902 for each dimension, all >0.70, and the test–retest reliability was 0.935. The correlation coefficient between the total score and each item was 0.585 to 0.875 (*p* < 0.01), the KMO value was 0.953, and the approximate value of the Barrett spherical test was *x* ^*2*^ = 6396.482 (*p* < 0.001), indicating good reliability and validity. Cronbach's α in this study was 0.954.

#### 3.2.3 Resilience Scale

The psychological resilience of clinical nurses was assessed using a Chinese version of the Resilience Scale. It was developed by Feng Suhong (2012). The scale included 4 dimensions, including cognition (6 items), social support (7 items), emotion (4 items), and will (5 items) with a total of 22 items. Each item was rated on a five‐point Likert scale (1 = highly inconsistent to 5 = highly consistent). The total score of the scale is 22 ~ 110 points, and the higher the score, the stronger the level of psychological resilience. Cronbach's α coefficient of the scale was 0.91 for the total scale and 0.78 ~ 0.86 for each dimension, all >0.70. The KMO value was 0.83, and the approximate value of the Barrett spherical test was *x* ^*2*^ = 1473.36 (*p* < 0.001), indicating good reliability and validity. Cronbach's α in this study was 0.880.

#### 3.2.4 Practice Environment Scale

The characteristics of the nursing practice environment in the hospital were measured by the Chinese version of the PES, as developed by Lake (2002) with Cronbach's α for each dimension ranging from 0.71 ~ 0.84. The Chinese version of PES was translated by Li and Lezhi (2011). The scale consisted of 5 dimensions and 31 items, including nurse participation in hospital affairs (9 items), nursing foundations for quality of care (10 items), nurse manager ability, leadership, and support of nurses (5 items), staffing and resource adequacy (4 items), and collegial nurse–physician relations (3 items). Each item was rated on a four‐point Likert scale (1 = strongly disagree to 4 = strongly agree). The total score was 31 to 124 points, and the higher the score, the better the nursing practice environment. In addition, the scale takes 2.5 points as the cutoff value for each item, with >2.5 points indicating that the respondents agree with the items investigated and <2.5 points indicating that the respondents disagree with the items investigated. Cronbach's α coefficient of the Chinese version of PES was 0.91 for the total scale and 0.67 ~ 0.79 for each dimension, and the test–retest reliability was 0.84. The correlation coefficient between the total score and each item was 0.62 to 0.88 (*p* < 0.01), the KMO value was 0.90, and the approximate value of the Barrett spherical test was *x* ^*2*^ = 4072.87 (*p* < 0.001), indicating good reliability and validity. Cronbach's α in this study was 0.905.

### 3.3 Data collection

The survey was carried out online through the “Questionnaire Star” platform (Wenjuanxing, http://www.wjx.cn), relying on WeChat (the largest Chinese social media platform). The questionnaires were conducted in Chinese. An illustration at the top of the questionnaire included the aim and significance of the study, and filling methods. The submission requirements for the “Questionnaire Star” were set to “participate in one time at most,” and “the questionnaire could only be submitted successfully if it was correctly filled out and there were no missing items.” We disseminated the recruitment information to the head nurses of different units in investigation hospital. Then, the head nurses announced the instructions for eligible nurses to participate in the investigation and gave the link to nurses via WeChat. Upon informed consent, each individual nurse was invited to participate in the study. The questionnaire would be submitted to the website backend once completed, and the head nurse did not have the authority to view it.

### 3.4 Statistical analysis

SPSS 25.0 was used to analyse the data. Continuous variables were tested for normality, which were all non‐normal distributions in this study and expressed as medians (interquartile ranges, IQRs); categorical variables were expressed as percentages. The Mann–Whitney U test was used to compare the non‐normal distribution data between two groups, and the Kruskal–Wallis H test was used to compare the non‐normal distribution data between multiple groups. Spearman's correlation analysis was used to analyse the correlation between nurses' moral courage, psychological resilience, and nursing practice environment. The influencing factors of clinical nurses' moral courage were analysed by hierarchical linear regression analysis. *p* < 0.05 was considered to be statistically significant.

### 3.5 Ethical consideration

This study was approved by the Ethics Committee of the First Affiliated Hospital of Chongqing Medical University; the authorization number is K2023‐045. In the consent form, we explained the background, purpose, process, and methods. Participants were informed of voluntary participation, anonymous responses, and confidentiality terms about their personal information. The research did not cause any harm to the research participants and followed the no‐harm principle. Participants may withdraw from the study at any time. The consent form was signed if the nurse agreed to participate in the study, and then, the survey was initiated.

## 4 RESULTS

### 4.1 General information

We screened 721 clinical nurses, and 593 participants met the inclusion criteria. However, 7 of the questionnaires were invalid due to obvious consistent options. Finally, there were 586 valid questionnaires, and the effective recovery rate was 98.82% (586/593). General information is shown in Table 1. Among the participants, 578 (98.6%) were female, 299 (51.0%) were 31–40 years old, and 113 (19.3%) participated in moral courage training.

**TABLE 1:** General information about clinical nurses (*n* = 586).

Variable	*n* (%)	Variable	*n* (%)
Gender		Employment form	
Female	578 (98.6)	Permanent employees	215 (36.7)
Male	8 (1.4)	Contractual employees	371 (63.3)
Age (years)		The prime shift in the past 6 months	
20 ~ 30	273 (46.6)	Day shift	201 (34.3)
31 ~ 40	299 (51.0)	Night shift	385 (65.7)
≥41	14 (2.4)	Highest degree	
Work experience (years)		Junior college degrees	79 (13.5)
1 ~ 4	61 (10.4)	Bachelor's degrees	494 (84.3)
5 ~ 9	291 (49.7)	Master's degrees	13 (2.2)
10 ~ 14	191 (32.6)	Have children	
≥15	43 (7.3)	Yes	426 (72.7)
Professional title		No	160 (27.3)
Junior nurses	87 (14.9)	Moral courage training	
Senior nurses	411 (70.1)	Yes	113 (19.3)
Chief nurses	88 (15.0)	No	473 (80.7)
Specialist nurses			
Yes	174 (29.7)		
No	412 (70.3)		

### 4.2 The score of moral courage, psychological resilience, and nursing practice environment of clinical nurses

The total score of moral courage was 79.00 (69.00, 91.00), the total score of psychological resilience was 78.00 (74.00, 83.00), and the total score of nursing practice environment was 91.00 (77.00, 102.00). The scores of each scale are shown in Table 2.

**TABLE 2:** The score of moral courage, psychological resilience, and nursing practice environment of clinical nurses [*n* = 586, *M* (P25, P75)].

Variable	No. of items	Score range	Score	Mean score of each item
Total NMCS	21	21 ~ 105	79.00 (69.00, 91.00)	3.76 (3.29, 4.33)
Moral integrity	7	7 ~ 35	27.00 (23.00, 31.00)	3.86 (3.28, 4.43)
Commitment to good care	5	5 ~ 25	17.00 (14.00, 21.00)	3.40 (2.80, 4.20)
Compassion and true presence	5	5 ~ 25	20.00 (17.00, 24.00)	4.00 (3.40, 4.80)
Moral responsibility	4	4 ~ 20	15.00 (12.00, 18.00)	3.75 (3.00, 4.50)
Total Resilience Scale	22	22 ~ 110	78.00 (74.00, 83.00)	3.55 (3.36, 3.77)
Cognition	6	6 ~ 30	19.00 (18.00, 21.00)	3.17 (3.00, 3.50)
Social support	7	7 ~ 35	26.00 (23.00, 29.00)	3.71 (3.29, 4.14)
Emotion	4	4 ~ 20	13.00 (12.00, 14.00)	3.25 (3.00, 3.50)
Will	5	5 ~ 25	20.00 (18.00, 22.00)	4.00 (3.60, 4.40)
Total PES	31	31 ~ 124	91.00 (77.00, 102.00)	2.94 (2.48, 3.29)
Nurse participation in hospital affairs	9	9 ~ 36	25.00 (20.00, 29.00)	2.78 (2.22, 3.22)
Nursing foundations for quality of care	10	10 ~ 40	31.00 (28.00, 36.00)	3.10 (2.80, 3.60)
Nurse manager ability, leadership, and support of nurses	5	5 ~ 20	15.00 (13.00, 17.00)	3.00 (2.60, 3.40)
Staffing and resource adequacy	4	4 ~ 16	11.00 (9.00, 13.00)	2.75 (2.25, 3.25)
Collegial nurse–physician relations	3	3 ~ 12	8.00 (7.00, 10.00)	2.67 (2.33, 3.33)

*Note*: NMCS, Nurses' Moral Courage Scale; PES, Practice Environment Scale.

### 4.3 Comparison of moral courage scores of clinical nurses with different characteristics

The clinical nurses were divided into different groups according to their gender, age, work experience, whether they were specialist nurses, professional title, employment form, the prime shift in the past 6 months, the highest degree, whether they had children, and whether they had participated in moral courage training, and the total score of moral courage was compared between each group. The results showed that the total score of nurses' moral courage with different professional titles, the highest degree, and whether they had children had no statistical significance (*p* > 0.05). There were statistically significant differences in the total score of moral courage among nurses of different genders, ages, work experience, whether they were specialist nurses, employment form, the prime shift in the past 6 months, and whether they had participated in moral courage training (*p* < 0.05). The results are shown in Table 3.

**TABLE 3:** Comparison of total moral courage scores of clinical nurses with different characteristics [*n* = 586, M (P25, P75)].

Variable	Total moral courage	Z/*χ* ^2^ value	*p* Value
Gender			
Male	92.00 (91.00, 95.00)	2.724	0.006^a^
Female	79.00 (69.00, 89.00)		
Age (years)			
21 ~ 30	79.00 (67.00, 98.00)	9.351	0.009^b^
31 ~ 40	79.00 (70.00, 89.00)		
≥41	87.00 (79.00, 103.00)		
Work experience (years)			
1 ~ 4	72.00 (59.00, 83.00)	9.099	0.028^b^
5 ~ 9	79.00 (71.00, 93.00)		
10 ~ 14	81.00 (66.00, 89.00)		
≥15	87.00 (77.00, 98.00)		
Specialist nurses			
Yes	85.00 (72.00, 91.00)	3.821	<0.001^a^
No	78.00 (69.00, 89.00)		
Employment form			
Permanent employees	81.00 (72.00, 94.00)	4.181	<0.001^a^
Contractual employees	72.00 (66.00, 87.00)		
The prime shift in the past 6 months			
Day shift	84.00 (70.00, 93.00)	3.383	0.001^a^
Night shift	77.00 (66.00, 88.00)		
Participated in the training of moral courage			
Yes	87.00 (76.00, 103.00)	4.821	<0.001^a^
No	79.00 (66.00, 89.00)		

^a^ Mann–Whitney U test.

^b^ Kruskal–Wallis H test.

### 4.4 Correlation analysis of psychological resilience, nursing practice environment, and moral courage

The results of Spearman's correlation analysis showed that the moral courage of clinical nurses was positively correlated with psychological resilience (rs = 0.441, *p* < 0.01) and nursing practice environment (rs = 0.505, *p* < 0.01). The results are shown in Table 4.

**TABLE 4:** Correlation analysis of psychological resilience, nursing practice environment, and moral courage (*n* = 586).

Variable	Moral integrity	Commitment to good care	Compassion and true presence	Moral responsibility	Total NMCS
Total Resilience Scale	0.429**	0.451**	0.314**	0.459**	0.441**
Cognition	0.147**	0.069	0.206**	0.054	0.147**
Social support	0.455**	0.477**	0.427**	0.462**	0.493**
Emotion	0.142**	0.137**	0.044	0.090*	0.106*
Will	0.421**	0.384**	0.378**	0.448**	0.438**
Total PES	0.469**	0.484**	0.444**	0.489**	0.505**
Nurse participation in hospital affairs	0.358**	0.425**	0.346**	0.425**	0.408**
Nursing foundations for quality of care	0.518**	0.456**	0.505**	0.547**	0.546**
Nurse manager ability, leadership, and support of nurses	0.457**	0.455**	0.419**	0.445**	0.476**
Staffing and resource adequacy	0.447**	0.486**	0.449**	0.456**	0.497**
Collegial nurse–physician relations	0.375**	0.398**	0.304**	0.353**	0.386**

Abbreviations: NMCS, Nurses' Moral Courage Scale; PES, Practice Environment Scale.

*p <0 .05; **p < 0.01.

### 4.5 Hierarchical linear regression analysis of influencing factors of nurses' moral courage

The total score of nurses' moral courage in this group was taken as the dependent variable, and the 7 variables with statistical significance in univariate analysis were taken as the control variables for preliminary analysis. The classified data were set as dummy variables and then put into Level 1 of the regression model; the correlation analysis of statistically significant 2 variables as independent variables was put into Level 2 of the regression model to conduct hierarchical linear regression analysis. Through the analysis of residuals, the Durbin–Watson value was 1.694, indicating data met linearity, independency, normal distribution, and equal variance. Collinearity diagnosis showed that the model tolerance was between 0.456 and 0.881, and the variance inflation factor value was between 1.135 and 2.193, meaning there was no multicollinearity among the nine independent variables (Jin, 2022; Kim, 2019; Liao et al., 2020). Hierarchical linear regression analysis showed that gender, employment form, and the prime shift in the past 6 months were the influencing factors at Level 1 (*p* < 0.05), and resilience and practice environment were the main influencing factors at Level 2, explaining 23.4% of the variance (Table 5).

**TABLE 5:** Hierarchical linear regression analysis on influencing factors of nurses' moral courage (*n* = 586).

Variable	*Β*	*SE*	*β*	*t*	*p*	95% CI
Level 1						
Gender						
Male	Ref.					
Female	−12.594	4.913	−0.092	−2.563	0.011	−22.244 ~ −2.944
Employment form						
Permanent employees	Ref.					
Contractual employees	−5.263	1.210	−0.160	−4.351	<0.001	−7.638 ~ −2.887
The prime shift in the past 6 months						
Day shift	Ref.					
Night shift	−4.020	1.282	−0.120	−3.136	0.002	−6.538 ~ −1.503
Level 2						
Psychological resilience	0.507	0.081	0.269	6.295	<0.001	0.349 ~ 0.665
Nursing practice environment	0.318	0.041	0.341	7.824	<0.001	0.238 ~ 0.397

*Note*: Ref., as the control group. Level 1, *R* ^*2*^ = 0.112, adjusted *R* ^*2*^ = 0.101, *F* = 10.332, *p* < 0.001; Level 2, *R* ^*2*^ = 0.345, adjusted *R* ^*2*^ = 0.335, *F* = 33.545, *p* < 0.001; Δ*R* ^*2*^ = 0.234.

## 5 DISCUSSION

As society continues to evolve and change, the physical and emotional pressures nurses suffer and the moral distress caused by conflicting professional values are extremely challenging, in which moral courage has been given a new role (Khodaveisi et al., 2021). Our study analysed the correlation between clinical nurses' psychological residence and nursing practice environment with moral courage, which has not been reported before. In this study, the total score of clinical nurses' moral courage was 79. 00 (69. 00, 91. 00), which was in the middle and upper level compared with the average score of the scale (52. 5); consistent with the findings of this study, other studies have shown that nurses, especially in central China and Finland, have reported high moral courage (Hu et al., 2022; Kleemola et al., 2020).

There are two possible explanations for the similar level of nurses' moral courage in this study: first, the participants investigated in this study were the nurses in the general hospital. As managers paid more attention to the training of nursing ethics education, nurses' ethical cultivation and moral courage have been improved. Second, clinical nurses in this group worked for an average of 5 to 14 years, accounting for 482 (82.3%) persons. As work experience grew, so did nurses' familiarity with nursing work content and environment, as well as their level of moral courage and brave behaviour (Huang et al., 2023; Khoshmehr et al., 2020). However, the moral courage level in northeast China was far lower than the results of this study conducted in southwest China, and the overall moral courage score of nurses is 42.00 vs. 79.00 points (Hong et al., 2023); Geographical and cultural differences may cause this.

Findings showed that the level of clinical nurses' moral courage was affected by gender, employment forms, and shifts (*p* < 0.05). The male nurses had higher moral courage level than female nurses in our study, while Pirdelkhosh et al. (2022) found that there were no statistically significant differences between male nurses and female nurses. Even more, our results are contrary to the conclusions of Hauhio et al.'s (2021) study. Given the study's small sample size of male nurses, our conclusion should be interpreted with caution (Hauhio et al., 2021).

In our study, permanent employees showed a higher level of moral courage than contractual employees, which was consistent with the results of Wang et al. (2019). The reason may be that permanent employees have a higher sense of job stability and better career advancement, and these positive psychological hints and emotional reinforcement can boost their moral level (Wang et al., 2019).

In this study, the moral courage level of nurses whose prime shift was the day shift was higher than that of middle and night shift nurses. It may be related to insufficient manpower resources, physical exhaustion, and other reasons for nurses in middle and night shifts, which cannot stimulate a higher level of moral courage.

The results showed that the psychological resilience of clinical nurses was in the middle level, and it was the main influencing factor of moral courage (*B* = 0.507, *p* < 0.001), indicating that the higher the nurses' psychological resilience, the higher their moral courage, which was consistent with the results of Abdollahi et al. (2021). Nurses have good psychological resilience in overcoming moral dilemmas, and fearlessly doing the proper action is at the heart of moral courage (Pajakoski et al., 2021). From the perspective of cognition, individuals with a high level of psychological resilience can positively and objectively evaluate the event itself and form correct cognition in a dilemma; from the perspective of behaviour, individuals with a high level of psychological resilience are more willing to actively find solutions to problems and adopt correct behaviour in difficulties (Iacoviello & Charney, 2014; Suhong, 2012). The same is true when individuals face moral dilemmas. Individuals with a high level of psychological resilience can form understanding and make judgements correctly, resulting in assertive moral courage behaviour (Khoshmehr et al., 2020). To be more specific, they can use adaptive skills to cope with pressure and effectively withstand the stressful environment of the hospital, so as to alleviate moral dilemmas (Abdollahi et al., 2021). To better accomplish clinical nursing work and guarantee the highest level of efficiency and quality, nurses should strengthen their psychological resilience, which will give them more flexibility in handling stressors, overcoming adversity, and turning it into hope (Rushton, 2023).

It was worth mentioning that Abdollahi et al.'s (2021) study showed that there was a statistically significant positive correlation between psychological resilience and moral courage; meanwhile, there was also a statistically significant positive correlation between psychological resilience and the subscale (moral agency, multiple values, the endurance of threat, going beyond compliance, and moral goals) of moral courage. The author further analysed the predictors of psychological resilience and found that the level of moral courage could explain 45% of the changes in psychological resilience, suggesting that the two are synergistic and influence each other.

The American Nurses Credentialing Center believes that a positive nursing practice environment is critical to improving the quality of care (Jingxia et al., 2022). The results of this study showed that the nursing practice environment was the main influential factor of nurses' moral courage (*B* = 0.318, *p* < 0.001), which means that the better the nursing practice environment, the higher their moral courage. Some studies have shown that the moral atmosphere, hierarchical structure, and teamwork atmosphere of the organization are necessary conditions for improving moral courage (Kleemola et al., 2020; Sadooghiasl et al., 2018). Poor communication, poor collaboration, and rejection from other professionals, such as physicians, affect the atmosphere of teamwork and may lead to job insecurity, consequently inhibiting moral courage (Pajakoski et al., 2021; Sadooghiasl et al., 2018). A good nursing practice environment or organizational care can increase the support for nurses' brave behaviour. For example, nurses have the opportunity to participate in moral decision‐making, dare to raise moral and ethical concerns, and receive positive discussions. The organization can establish a virtuous loop and boost moral courage when these actions are permitted and affirmed by the organization (Pajakoski et al., 2021).

In this study, the average score of the nursing practice environment was 2.94, which was in the upper and middle level, similar to the study of Membrillo‐Pillpe et al. (2023). However, all subscales scored below 3.0 except for nursing foundations for quality of care and nurse manager ability. Based on the general background of China, workforce shortage and poor organizational communication are two challenges affecting the nursing practice environment, and taking appropriate measures to improve the nursing practice environment from the root is essential to creating a positive ethical climate (Hu et al., 2022).

## 6 LIMITATIONS

This study also has some limitations. First, the sample size of this study comes from only one large hospital, so the sample range was limited. Second, the regression model only showed that moral courage was affected by psychological resilience and nursing practice environment, and cohort studies are needed to verify the causal relationship. Third, only part of the inner factors of the TLC model were analysed in this study and the results of hierarchical linear regression analysis showed that the adjusted coefficient of determination was 0.234, which indicated that the investigated influencing factors were not comprehensive. In the future, more research into potential predictors of moral courage is needed to offer a scientific foundation for the development of intervention methods for moral courage.

## 7 CONCLUSION

Moral courage is considered one of the nursing profession's core virtues, and all nurses need moral courage. The results of this study showed that the moral courage of clinical nurses was at a middle and upper level, with room for improvement, and was related to five factors. Psychological resilience and nursing practice environment, as core factors, were closely related to the moral courage level of nurses. These findings provided some reference for formulating effective measures to alleviate nurses' moral dilemmas and improve their moral courage.

## 8 RELEVANCE FOR CLINICAL PRACTICE

To help hospital managers improve the moral level of clinical nurses further, we recommend focusing on nurses' mental health and improving the nursing practice environment as part of the hospital policy. The following strategies should be considered to improve the moral courage level of nurses: (1) Carry out education training and practice. Nursing managers can rectify nurses' perceptions of moral courage by delivering moral courage education or recreating real‐life ethical circumstances. For example, Edmonson's (2015) CODE model, which is built on courage, obligations, danger, and expression, can be used to design courses and train nurses, which can be used as a theoretical basis for curriculum design and can effectively improve the level of moral courage. In addition, managers who play good role models may encourage nurses to follow suit and enhance their moral courage (Sadooghiasl et al., 2018). (2) Create a good nursing practice environment. Actively improve the internal and external conditions of the organization, such as changing leadership style, eliminating hierarchy, enhancing psychological empowerment, smoothing communication and feedback channels, and building a patient safety culture, to create a safe, open, and supportive environment for ethical behaviour. (3) Pay attention to the psychological emotions of nurses. (4) Understand the nurses' personality characteristics and emotional changes and provide system support for female nurses, contractual employees, and middle and night shift nurses. Medical decision‐makers should take note of these measures, as they have the potential to enhance the working conditions, mental health, and morale of nurses while also lowering the rate of nursing staff turnover and improving the quality of nursing.

## AUTHOR CONTRIBUTIONS

Zhang Ruixin: Writing – original draft; writing – review and editing; methodology; and data curation. He Shan: Investigation. Tang Yongli: Funding acquisition; project administration; resources; and supervision. Chen Jie: Supervision; resources; project administration; and funding acquisition. Chen Qianzhu: Supervision; resources; and investigation. Wang Xue: Data curation; writing – review and editing; and writing – original draft.

## FUNDING INFORMATION

The author(s) disclosed receipt of the following financial support for the research, authorship, and/or publication of this article: this study was funded by the Municipal Education Commission, Chongqing, China (decision 13.5.2020; no. 20SKGH031 for the period 2019–2022).

## CONFLICT OF INTEREST STATEMENT

The author(s) declared no potential conflicts of interest with respect to the research, authorship, and/or publication of this article.

## OTHERS

For a more detailed review in the context and for a full discussion of our findings in the discussion, we have more than 25 references. The statistics were checked prior to submission by the expert statistician, Fei Long, 13883575719@163.com.

## Data Availability

The datasets generated during and/or analysed during the current study are available from the corresponding author upon reasonable request.
